# Successful Treatment of Refractory Methicillin-Sensitive Staphylococcus aureus Osteomyelitis With Ceftaroline Fosamil in a Diabetic Patient

**DOI:** 10.7759/cureus.88049

**Published:** 2025-07-16

**Authors:** Sarah Baroud, Fatima Alhammadi, Fatima Alhashimi, Jawad Elhaout, Hasanain Al-Shakerchi

**Affiliations:** 1 Internal Medicine, Mediclinic Parkview Hospital, Dubai, ARE; 2 Internal Medicine, Mohammed Bin Rashid University of Medicine and Health Sciences, Dubai, ARE

**Keywords:** ceftaroline fosamil, diabetes mellitus, methicillin-sensitive staphylococcus aureus, osteomyelitis, salvage therapy

## Abstract

Osteomyelitis is a challenging complication in patients with diabetes mellitus, often necessitating prolonged antimicrobial therapy and surgical intervention. We present the case of a 70-year-old man with poorly controlled type 2 diabetes who developed recurrent diabetic foot osteomyelitis and vertebral osteomyelitis caused by methicillin-susceptible *Staphylococcus aureus* (MSSA). Despite receiving multiple courses of IV antibiotics and undergoing surgical debridement, the patient experienced persistent bacteremia without clinical improvement. Ceftaroline fosamil, a fifth-generation cephalosporin with activity against both MSSA and methicillin-resistant *S. aureus*, was initiated. Following treatment, the patient achieved bacteriologic clearance and marked clinical improvement. This case underscores the potential role of ceftaroline in managing complex, refractory osteomyelitis, particularly when conventional therapies fail or are limited by resistance, adverse effects, or availability.

## Introduction

Osteomyelitis is a serious bone infection characterized by inflammation, tissue necrosis, and bone destruction, often worsened by poor vascularity and an impaired immune response. In individuals with diabetes mellitus, it is a common and challenging complication, affecting up to 20% of diabetic foot infections and significantly increasing the risk of lower limb amputation. *Staphylococcus aureus*, including both methicillin-susceptible (MSSA) and methicillin-resistant (MRSA) strains, is the most frequently isolated pathogen. While acute osteomyelitis may respond to antimicrobial therapy alone, chronic or hardware-associated cases typically require prolonged IV antibiotics, often in conjunction with surgical debridement. In diabetic patients, reduced bone perfusion further complicates treatment by limiting antibiotic penetration and decreasing the likelihood of achieving therapeutic concentrations at the site of infection. Emerging clinical data suggest that ceftaroline fosamil, a fifth-generation cephalosporin, may be effective in the off-label treatment of osteomyelitis, particularly for MRSA and MSSA infections in complex patient populations with diabetes or peripheral artery disease (PAD), especially when standard therapies have failed or are not feasible [1-5].

Here, we report a case of severe, refractory MSSA osteomyelitis in a diabetic patient successfully treated with ceftaroline fosamil.

## Case presentation

A 70-year-old retired Emirati male with poorly controlled type 2 diabetes was brought to the hospital in a wheelchair, presenting with a three-day history of persistent lower back pain and left foot pain with swelling. His symptoms began approximately two weeks after returning from Thailand, where he had received treatment for a left diabetic foot abscess; however, no treatment records were available.

Over the past year, he had been managed by the vascular surgery team for multiple diabetic foot complications, including foot ulcers, abscesses, osteomyelitis, and Charcot foot deformity. He had no implanted hardware or prosthetic joints. His treatment history included wound care, surgical debridement, and prolonged courses of IV antibiotics. He had a 20-year history of type 2 diabetes mellitus, managed with a basal-bolus insulin regimen, though adherence had been poor.

On examination, his vital signs were within normal limits. His BMI was 28.40 kg/m² (reference range: 18.5-24.9 kg/m²). He appeared to be in moderate pain. The left foot showed a Charcot deformity and was swollen, tender, erythematous, and warm to the touch. An open, non-draining wound was noted on the plantar aspect of the left midfoot, along with a palpable, fluctuating collection extending from the dorsum to the plantar surface. Pedal and popliteal pulses were palpable bilaterally. Neurological examination findings were consistent with diabetic peripheral neuropathy. Lumbar spine examination revealed tenderness in the right paraspinal region. Cardiac, pulmonary, abdominal, and other neurological examinations were unremarkable.

Initial laboratory results were notable for leukocytosis with neutrophil predominance (white blood cell count: 13.9 × 10³/μL and neutrophils: 86.7%), elevated inflammatory markers (C-reactive protein: 222 mg/L, erythrocyte sedimentation rate: 79 mm/hr, and procalcitonin: 0.76 ng/mL), and poor glycemic control (HbA1c: 8.1% and fasting glucose: 11.7 mmol/L). Additional findings included mild anemia (hemoglobin: 12.6 g/dL) and hypoalbuminemia (albumin: 25 g/L), with normal renal and liver function (Table 1).

**Table 1:** Laboratory results at the time of admission

Lab marker	Finding	Reference range
White blood cell count	13.9 × 10³/μL	4.0-11.0 × 10³/μL
Neutrophils	86.75%	43.5-73.5%
Lymphocytes	4.97%	15.2-43.3%
Red blood cell count	6.09 × 10⁶/μL	4.50-5.90 × 10⁶/μL
Hemoglobin	12.6 g/dL	13.0-17.5 g/dL
Platelet count	177 × 10³/μL	150-450 × 10³/μL
C-reactive protein	222 mg/L	0-5 mg/L
Procalcitonin	0.76 ng/mL	<0.05 ng/mL
Erythrocyte sedimentation rate	79 mm/hr	3-55 mm/hr
Creatinine	69.1 μmol/L	62.0-106.0 μmol/L
Estimated glomerular filtration rate	96 mL/min/1.73 m²	>60 mL/min/1.73 m²
HbA1c	8.10%	4.0-5.6%
Albumin	25 g/L	32-46 g/L

**Figure 1:**
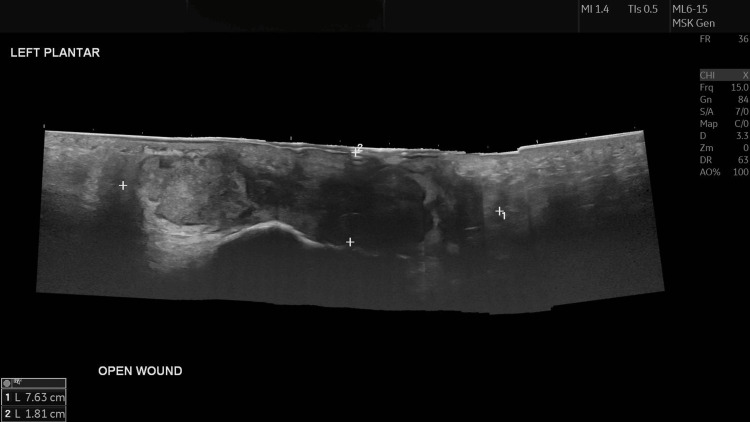
Ultrasound of the left foot demonstrating a 7.6 cm × 1.8 cm × 2.5 cm heterogeneous collection

Ultrasound of the left foot revealed a heterogeneous collection measuring 7.6 cm × 1.8 cm × 2.5 cm in the subcutaneous plane, extending deep to contact the underlying bone, suggestive of recurrent osteomyelitis (Figure 1).

Blood cultures were obtained, and the patient was admitted to the medical ward. Empirical IV piperacillin/tazobactam (4.5 grams every six hours) was initiated, along with tight glycemic control using insulin.

**Figure 2:**
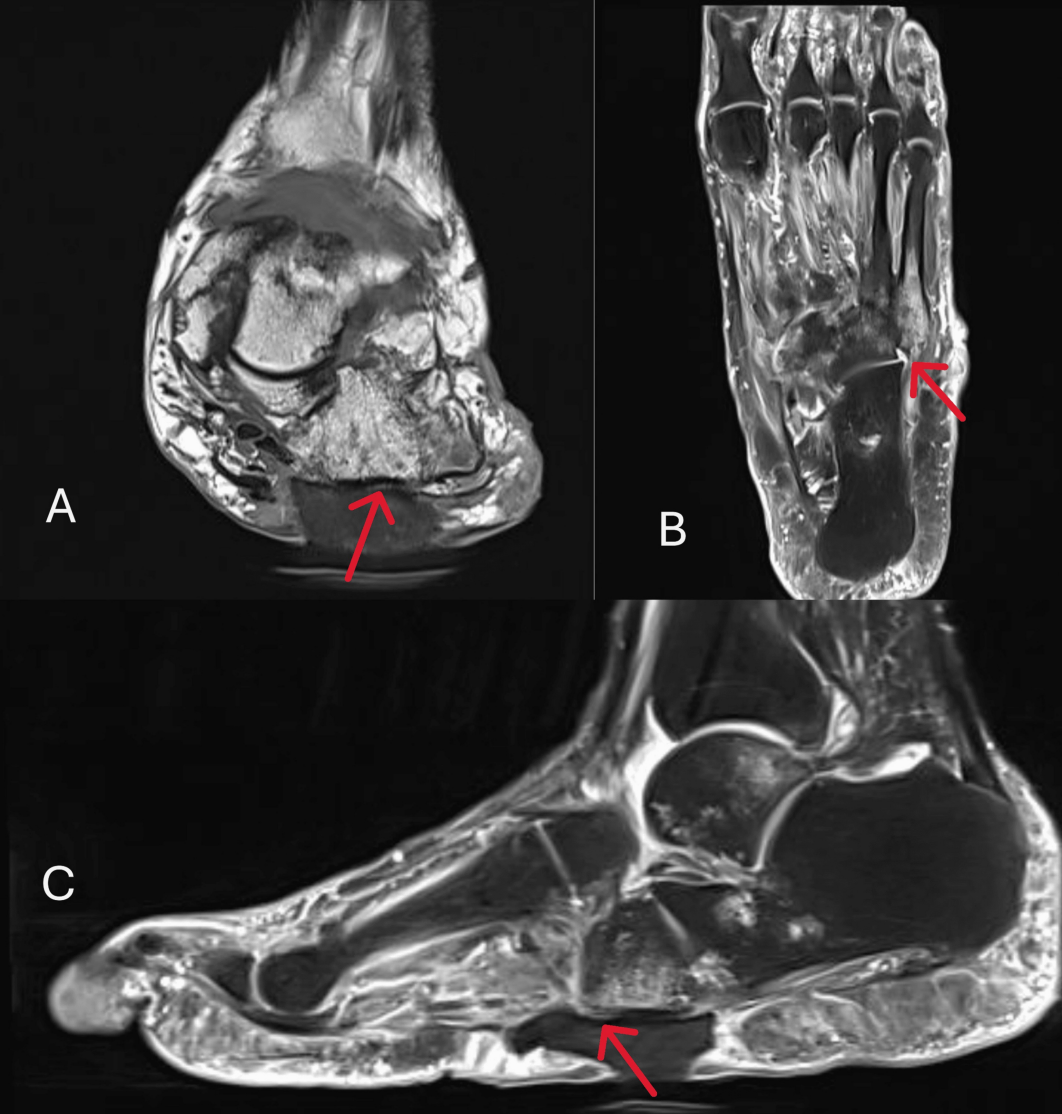
MRI of the left foot — (A) axial, (B) coronal, and (C) sagittal views — demonstrating a gas-containing soft tissue defect at the plantar lateral aspect of the midfoot, with the deep margin of the defect bordering the cuboid bone, and pronounced marrow edema in the cuboid and fifth metatarsal base with corresponding low T1 signal intensity and loss of cortical definition, consistent with osteomyelitis The arrows indicate the affected cuboid and base of the fifth metatarsal, showing marrow edema and cortical erosion in all three planes.

The vascular surgery team was consulted and proceeded with incision and drainage of the left diabetic foot abscess, debridement of necrotic tissue, and placement of a wound vacuum-assisted closure (VAC). An MRI of the left foot was subsequently performed to further assess bone involvement. It demonstrated Charcot foot deformity with findings suggestive of acute-on-chronic osteomyelitis involving the cuboid bone and the base of the fifth metatarsal, without evidence of an organized or drainable soft tissue collection (Figure 2).

Blood cultures, pus cultures, and bone biopsy cultures all grew MSSA with an oxacillin-sensitive minimum inhibitory concentration (MIC) of 0.5 µg/mL.

The patient initially received three doses of IV piperacillin/tazobactam, which was then de-escalated to IV cefazolin 2 grams every eight hours following identification of MSSA. After determination of the oxacillin MIC and in consultation with the hospital microbiologist, cefazolin was switched to IV flucloxacillin 2 grams every six hours, two days into cefazolin therapy, with a plan to complete a two-week course.

**Figure 3:**
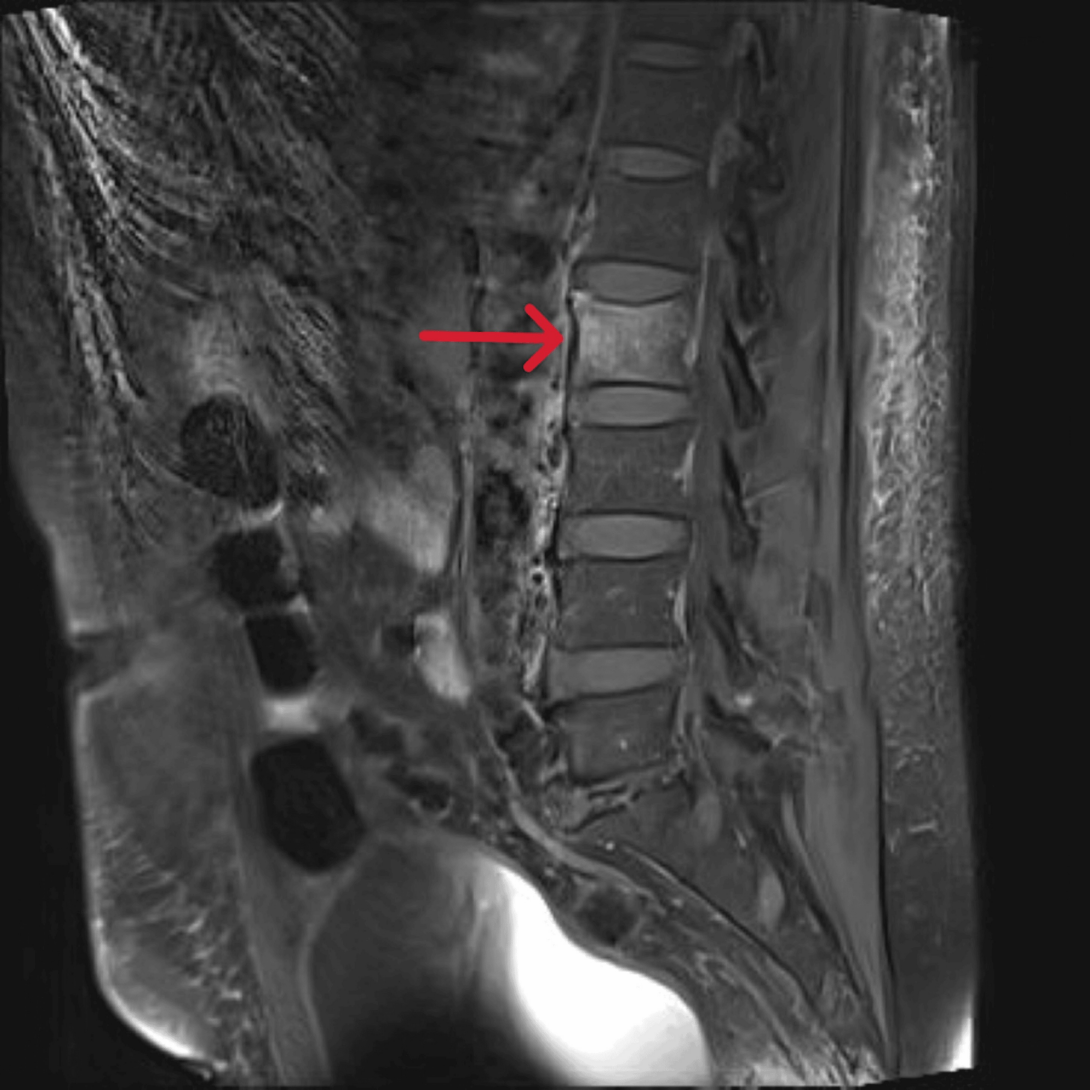
Contrast-enhanced MRI of the lumbosacral spine demonstrating increased signal intensity in the L2 vertebral body, with mild collapse of the superior endplate, consistent with vertebral osteomyelitis The arrow highlights the affected L2 vertebra with marrow changes.

Given the patient’s back pain and ongoing bacteremia, a contrast-enhanced MRI of the lumbosacral spine was performed. It revealed findings consistent with osteomyelitis of the L2 vertebral body, including marrow edema, endplate erosion, and adjacent soft tissue inflammation, without evidence of discitis or abscess formation. Incidental degenerative changes were also noted at L5-S1, with possible L5 nerve root compression (Figure 3).

After nine days of inpatient treatment, the patient remained hemodynamically stable and afebrile. The left foot wound appeared clean, with no discharge, and the VAC dressing was changed regularly. Inflammatory markers showed a downward trend (Table 2); however, blood cultures - repeated every 48 hours - remained persistently positive. In addition, the left ankle remained warm to the touch, and the patient continued to report axial low back pain and tenderness without distal motor or sensory deficits, despite ongoing physiotherapy and analgesia.

**Table 2:** Laboratory results on day 9 of antibiotic therapy

Lab marker	Admission day	On day 9 of antibiotics	Reference range
White blood cell count	13.9 × 10³/μL	8.9 × 10³/μL	4.0-11.0 × 10³/μL
Neutrophils	86.75%	78.24%	43.5-73.5%
Lymphocytes	4.97%	14.43%	15.2-43.3%
C-reactive protein	222 mg/L	73.2 mg/L	0-5 mg/L
Procalcitonin	0.76 ng/mL	0.06 ng/mL	<0.05 ng/mL

A repeat contrast-enhanced MRI of the lumbosacral spine showed progression of L2 vertebral osteomyelitis, while follow-up MRI of the left foot confirmed ongoing osteomyelitis without any drainable collections. A spinal surgeon was consulted and recommended continuing antibiotic therapy for at least six to eight weeks, considering the use of a hyperextension brace to support the L2 vertebra, and reserving surgical intervention for cases where medical therapy failed or there was evidence of neurological compromise or bony instability. A repeat spinal MRI was advised in two weeks, or earlier if clinically indicated.

In light of these findings, IV vancomycin was added to the antibiotic regimen, with dosing adjusted based on creatinine clearance and trough levels, targeting a range of 10-15 µg/mL. Due to persistent bacteremia, a transthoracic echocardiogram (TTE) was performed, which showed no evidence of valvular disease or endocarditis. However, blood cultures remained persistently positive for MSSA despite 17 days of inpatient antibiotic therapy, with no change in MIC values. Given the limited sensitivity of TTE in detecting endocarditis in MSSA bacteremia, the cardiology team recommended a transesophageal echocardiogram, which the patient declined. As an alternative, serial weekly TTEs were performed, all of which remained unchanged.

During the course of treatment, the patient developed a drug-induced skin rash, which the dermatology team attributed to IV vancomycin. Concurrently, the left foot showed clinical worsening, with increasing swelling and erythema. On day 17 of treatment, vancomycin was discontinued, with subsequent resolution of the rash, and was replaced with IV teicoplanin (administered as three loading doses of 10-12 mg/kg, followed by 1000 mg every 24 hours), in consultation with the infectious diseases team. Trough levels were monitored on day 5, with a target range of 20-40 µg/mL.

A follow-up MRI of the left foot showed increased marrow edema in the cuboid bone and persistent edema in the base of the fifth metatarsal, now extending more distally. Additionally, new patchy marrow edema was observed diffusely involving the talar dome, distal fibula, and distal tibia, centered at the medial malleolus. C-reactive protein levels were slowly rising (Table 3).

**Table 3:** Laboratory results on day 17 of antibiotic therapy

Lab marker	Admission day	Day 9 of antibiotics	Day 17 of antibiotics	Reference range
White blood cell count	13.9 × 10³/μL	8.9 × 10³/μL	6.0 × 10³/μL	4.0-11.0 × 10³/μL
Neutrophils	86.75%	78.24%	74.73%	43.5-73.5%
Lymphocytes	4.97%	14.43%	13.61%	15.2-43.3%
C-reactive protein	222 mg/L	73.2 mg/L	78.2 mg/L	0-5 mg/L
Procalcitonin	0.76 ng/mL	0.06 ng/mL	0.04 ng/mL	<0.05 ng/mL

The frequency of IV flucloxacillin was increased to 2 grams every four hours. The vascular surgery team was reconsulted and advised extending antibiotic therapy for at least three months, with consideration of below-the-knee amputation if the infection remained unresolved or if the patient developed clinical sepsis.

After nearly four weeks of IV antibiotic treatment, blood cultures remained positive, and the patient’s condition deteriorated. He developed new symptoms, including fever, worsening back pain, and sloughing of the left foot wound, which required de-sloughing dressings. A follow-up MRI of the spine showed progression of L2 osteomyelitis, now with a pathological fracture and a rim of epidural enhancement posterior to the L2 vertebral body, suggestive of early epidural involvement. No epidural collection was identified.

A thoracolumbar brace was applied by the spine surgeon. The vascular surgery team again recommended below-the-knee amputation, which the patient strongly declined. The infectious diseases team proposed a PET scan to assess for occult infection, but the patient refused. Due to the unavailability of alternative antibiotics such as nafcillin, oxacillin, and daptomycin in Dubai, options for optimizing antimicrobial therapy were limited. Given these constraints, the infectious diseases team recommended switching from IV flucloxacillin to IV ceftaroline 600 mg every eight hours while continuing teicoplanin.

Two days after the switch, blood cultures turned negative for the first time and remained negative thereafter. Over the following days, the patient’s back pain improved, his temperature stabilized, and the left foot wound remained dry, with resolution of erythema and no signs of abscess or discharge. Laboratory markers continued to show improvement.

A peripherally inserted central catheter (PICC) was placed to facilitate ongoing outpatient antibiotic therapy. The patient was scheduled to complete a 12-week course of antibiotics, starting from the first negative blood culture. This included 14 days of IV teicoplanin administered concurrently with six to eight weeks of IV ceftaroline, followed by oral antibiotics tailored to clinical response and local drug availability.

The vascular surgery team recommended twice-weekly wound dressing changes and the use of an immobilizer boot. They also cautioned that, even with successful medical therapy, the risk of relapse remained high and future major amputation was likely. The overall prognosis was considered poor, given the severity of the disease.

Ultimately, the patient chose to continue treatment abroad and was lost to follow-up.

## Discussion

Osteomyelitis is a bone infection characterized by inflammation that can lead to progressive tissue necrosis and destruction. This damage is often driven by swelling that compromises local blood flow, resulting in ischemia. Over time, these poorly perfused or avascular regions hinder both antibiotic penetration and immune cell delivery, making infection clearance difficult. In patients with diabetes mellitus, osteomyelitis presents an even greater challenge, occurring in up to 20% of diabetic foot infections and significantly increasing the risk of lower extremity amputation. Impaired wound healing, peripheral neuropathy, and PAD all contribute to the complexity and chronicity of infection [1].

*S. aureus* - including both MSSA and MRSA strains - is the most frequently isolated pathogen in osteomyelitis, accounting for approximately 40% of cases [1]. It commonly colonizes the skin, nasal passages, and gastrointestinal tract, with up to 80% of individuals being asymptomatic carriers. However, colonization can serve as a precursor to *S. aureus* bacteremia, which affects more than 50 individuals per 100,000 population annually [2]. This risk is significantly higher in people with diabetes, who face not only increased incidence but also greater disease severity and mortality [3]. Additional risk factors include frequent healthcare exposure and immunocompromised states. Clinical presentations range from asymptomatic bacteremia identified incidentally to prolonged infections complicated by embolic dissemination. Despite appropriate antimicrobial therapy and source control, the mortality rate for *S. aureus* bacteremia remains high, approximately 20-30% in developed countries [2].

Acute osteomyelitis often responds to antimicrobial therapy alone, while chronic cases, particularly those involving prosthetic material, typically require a combination of medical and surgical management. Standard treatment involves four to six weeks of IV antibiotics, with reported cure rates between 60% and 90%. In selected patients, oral agents with high bioavailability may be appropriate alternatives [1]. For MSSA bacteremia, anti-staphylococcal penicillins or cefazolin are preferred due to better clinical outcomes compared to broad-spectrum agents. In contrast, MRSA infections are typically treated with IV vancomycin or daptomycin, depending on the clinical context. While linezolid is effective for MRSA pneumonia, skin and soft tissue infections, or meningitis, its use in bacteremia has been linked to poorer outcomes. Similarly, agents such as trimethoprim-sulfamethoxazole, tetracyclines, clindamycin, and oral beta-lactams are generally not recommended for bacteremia due to limited supporting evidence [2].

Ceftaroline fosamil, a fifth-generation cephalosporin, has emerged as a promising off-label option for the treatment of refractory osteomyelitis. It has broad-spectrum activity against Gram-positive organisms - including MRSA and MSSA - and select Gram-negative pathogens. Its bactericidal effect is mediated through inhibition of bacterial cell wall synthesis by high-affinity binding to penicillin-binding proteins (PBPs), particularly PBP2a and PBP2x - key targets in MRSA and multidrug-resistant *Streptococcus pneumoniae *[2,4]. Although currently approved by the FDA for acute bacterial skin and skin structure infections and community-acquired bacterial pneumonia, increasing clinical evidence supports its broader therapeutic potential. In the CAPTURE study, a large multicenter retrospective analysis, ceftaroline achieved a 92.7% clinical success rate in patients with osteomyelitis caused by MRSA or MSSA, including those with comorbid diabetes and PAD [1,4]. A separate case series reported similar success rates in vertebral and non-vertebral osteomyelitis (57% vs. 60%), despite the vertebral group requiring longer hospital stays and extended treatment durations [5].

Ceftaroline is particularly useful in cases where standard therapies fail or are not viable due to intolerance or limited availability. It provides reliable bone penetration and a favorable safety profile and can be administered as monotherapy or in combination regimens. A 2022 case series from Buenos Aires described its use as salvage therapy in five adults with complicated MSSA bacteremia unresponsive to prolonged cefazolin treatment (median duration: 10 days). These patients had deep-seated infections - including endocarditis and metastatic foci - and all achieved microbiological clearance and clinical cure after switching to ceftaroline. It was used as monotherapy in four cases and in combination with daptomycin in one, reinforcing its potential role in managing refractory MSSA bacteremia, particularly when there is metastatic involvement [6]. Additional studies also support its synergistic use with agents like daptomycin or β-lactams to enhance bacterial eradication in persistent *S. aureus* infections [7,8].

Despite these advantages, ceftaroline has several limitations, including the need for multiple daily IV doses, the absence of an oral formulation, limited Gram-negative coverage, and the requirement for renal dose adjustment. Emerging resistance further highlights the importance of judicious use and the need for additional prospective studies [4,9,10].

In the present case, conventional therapy with IV flucloxacillin failed to control persistent MSSA bacteremia in the context of extensive diabetic foot infection and concurrent vertebral osteomyelitis. Vancomycin was discontinued due to a drug-induced rash, and alternative agents such as daptomycin and oxacillin were not locally available.

Given these challenges, ceftaroline fosamil was chosen for its potent activity against MSSA, favorable safety and pharmacokinetic profile, and emerging evidence supporting its use in diabetic foot and vertebral osteomyelitis. Additionally, it was administered alongside teicoplanin to provide early dual coverage for possible MRSA or undetected resistant organisms, despite culture-confirmed MSSA, due to the chronicity of infection and previous antibiotic exposure. This strategy was employed to maximize early treatment efficacy in a complex, refractory clinical scenario. The patient subsequently achieved rapid bacteriological clearance and marked clinical improvement, ultimately avoiding surgical intervention or amputation.

This outcome highlights the effectiveness of ceftaroline as a valuable salvage therapy in the management of complex, treatment-refractory infections. It also demonstrates how ceftaroline can be strategically incorporated into treatment regimens based on clinical judgment, microbiologic data, and logistical considerations.

## Conclusions

Ceftaroline fosamil has shown strong clinical effectiveness in treating Gram-positive osteomyelitis, particularly in high-risk populations such as individuals with diabetes, PAD, or prior antibiotic exposure. Real-world data, case series, and retrospective studies support its role as a viable therapeutic option, especially in infections caused by MRSA and MSSA. While randomized controlled trials are still lacking, ceftaroline has been successfully used as salvage therapy in cases where standard treatments failed or were poorly tolerated. Further prospective studies are needed to better define its clinical indications and optimize its use. Overall, ceftaroline fosamil represents a valuable addition to the antimicrobial arsenal for managing challenging osteomyelitis cases, including refractory MSSA infections in diabetic patients, as demonstrated in this case.

## References

[1] Johnson LB, Ramani A, Guervil DJ (2019). Use of ceftaroline fosamil in osteomyelitis: CAPTURE study experience. BMC Infect Dis.

[2] Lam JC, Stokes W (2023). The golden grapes of wrath - Staphylococcus aureus bacteremia: a clinical review. Am J Med.

[3] Bryan CS, Reynolds KL, Metzger WT (1985). Bacteremia in diabetic patients: comparison of incidence and mortality with nondiabetic patients. Diabetes Care.

[4] Shirley DA, Heil EL, Johnson JK (2013). Ceftaroline fosamil: a brief clinical review. Infect Dis Ther.

[5] Lalikian K, Parsiani R, Won R, Chang E, Turner RB (2018). Ceftaroline for the treatment of osteomyelitis caused by methicillin-resistant Staphylococcus aureus: a case series. J Chemother.

[6] Obed MN, Toresani I, Mykietuk A, Nannini EC (2022). Ceftaroline as salvage therapy for methicillin susceptible Staphylococcus aureus complicated bacteremia. Medicina (B Aires).

[7] Sakoulas G, Moise PA, Casapao AM (2014). Antimicrobial salvage therapy for persistent staphylococcal bacteremia using daptomycin plus ceftaroline. Clin Ther.

[8] Warren EF, Crocker RJ, Tabor B (2023). Successful use of nafcillin and ceftaroline combination therapy for persistent MSSA bacteraemia and endocarditis: a case series. JAC Antimicrob Resist.

[9] Frampton JE (2013). Ceftaroline fosamil: a review of its use in the treatment of complicated skin and soft tissue infections and community-acquired pneumonia. Drugs.

[10] Abate G, Wang G, Frisby J (2022). Ceftaroline: systematic review of clinical uses and emerging drug resistance. Ann Pharmacother.

